# Synthesis and Gas Transport Properties of Poly(2,6-dimethyl-1,4-phenylene oxide)–Silica Nanocomposite Membranes

**DOI:** 10.3390/membranes8040125

**Published:** 2018-12-04

**Authors:** Golnaz Bissadi, Thiago Melo Santos, Boguslaw Kruczek

**Affiliations:** Department of Chemical and Biological Engineering, University of Ottawa, 161 Louis-Pasteur, Ottawa, ON K1N 6N5, Canada; gbis013@gmail.com (G.B.); thiagomelosts@gmail.com (T.M.S.)

**Keywords:** nanocomposite membranes, gas separation, poly(2,6-dimethyl-1,4-phenylene oxide), tetraethylorthosilicate

## Abstract

The emulsion polymerized mixed matrix (EPMM) method is a new approach to prepare nanocomposite membranes, in which inorganic nanoparticles are synthesized in situ at the interface of a dispersed aqueous phase in a continuous phase of polymer solution. In this paper, we report the synthesis and characterization of poly(2,6-dimethyl-1,4-phenylene oxide) (PPO)-based EPMM membranes, in which silica nanoparticles are synthesized by the polymerization of tetraethylorthosilicate (TEOS) in the presence of two different co-solvents, ethanol and acetone, which are soluble in both the aqueous phase and the polymer solution. The EPPM membranes prepared in the presence of acetone show greater conversions of TEOS and a different structure of the synthesized silica nanoparticles compared to the EPMM membranes prepared in the presence of ethanol. The former membranes are both more permeable and more selective for O_2_/N_2_ and CO_2_/CH_4_. Both types of EPMM membranes are more permeable than the reference PPO membranes. However, while their O_2_/N_2_ selectivity is practically unchanged, their CO_2_/CH_4_ selectivity is decreased compared to the reference PPO membranes.

## 1. Introduction

In the last two decades, gas separation by polymeric membranes is one of the fastest-growing branches of membrane technology [1,2,3,4]. Polymeric membranes are advantageous due to their flexibility, processability, ability to withstand high pressures, and lower energy consumption compared to other unit operations [5,6]. On the other hand, their performance is characterized by a trade-off between the gas permeability and the gas selectivity; their selectivity is generally low, and they cannot operate at high temperatures [6,7,8]. Inorganic membranes overcome some of these limitations, but they are expensive and difficult to fabricate owing to their fragile structures [9]. The mixed matrix membranes (MMM), which are sometimes referred to as hybrid membranes and more recently as nanocomposite membranes, are composed of inorganic nanoscale particles dispersed in an organic polymer matrix. These are seen as a way of combining the advantages of inorganic materials with those of organic polymers, in particular to solve the trade-off problem [6,10,11,12].

There are many different inorganic materials that have been considered for the preparation of nanocomposite membranes [13]. Silica nanoparticles, which are commercially available and easy to synthesize at room temperature [14,15], are among the most commonly used materials. The preparation of uniform silica particles that were 50–2000 nm in diameter was first reported by Stöber and Fink through the hydrolysis and condensation of tetraethylorthosilicate (TEOS) in the mixture of ethanol, water, and ammonia, which is referred to as a sol-gel method [16]. Since then, diverse studies on the Stöber method have been carried out. Generally, the sol-gel process involves two main steps, hydrolysis and condensation of metal alkoxides, Si(OR)_4_, in the presence of an acid or base catalyst [17,18]. Alternatively, the synthesis of silica from TEOS can also be carried out in water/oil (W/O) microemulsions, which offer attractive possibilities for the preparation of a variety of nanomaterials [19,20,21,22,23,24]. The main advantage of this method is the synthesis of much smaller colloids—25–70 nm in diameter—compared to the silica obtained by the Stöber method [25]. Although a basic catalyst leads to monodispersed spherical silica particles, the corresponding dimensions are above 100 nm, i.e., in a micrometer range [20,26]. On the other hand, if an acid catalyst is used, a transparent sol is obtained with particles generally below 100 nm [27]. Therefore, the polymer/silica nanocomposites to be used in nanocomposite membranes are prepared by using an acid-catalysis [27,28,29].

Poly(2,6-dimethyl-1,6-phenylene oxide) (PPO) is a commercially available glassy polymer with a high (>210 °C) glass transition temperature, and excellent mechanical and thermal properties. PPO is characterized by its high permeability to gases, which is attributed to the absence of polar groups in the polymer backbone, and moderate gas selectivity [30,31,32,33,34,35]. The applications to form nanocomposite membranes with silica particles are limited to chemically modified PPO [36,37,38,39,40]. This is because the synthesis of silica-PPO nanocomposite membranes by the direct dispersion of nanoparticles in a polymer solution, which is also referred to as solution blending [26,41], leads to the agglomeration of nanoparticles and ultimately to phase separation [42]. To improve the dispersion of nanoparticles in the polymer matrix, nanocomposite membranes are also formed by a sol-gel method, in which nanoparticles are synthesized from a precursor dissolved in a polymer solution [43]. However, this method is not suitable for the synthesis of silica-PPO nanocomposite membranes, because typical silica precursor solutions are only partly miscible with common PPO solvents.

To alleviate this limitation, we have developed an emulsion polymerized mixed matrix (EPMM) method [42,44,45]. Similar to the sol-gel method, the EPMM method employs a silica precursor, but the polymerization reaction takes place in an aqueous phase, which is dispersed in a continuous phase of polymer solution. The first generation PPO-based EPMM membranes were prepared using a surfactant (n-octanol) to facilitate the transport of silica precursor, tetraethylorthosilicate (TEOS), from the continuous phase of the polymer solution to the dispersed aqueous phase containing a catalyst that is necessary for the polymerization of TEOS to occur [44]. However, the gas transport properties of the first generation EPPM membranes were not improved compared to the neat PPO membrane [44]. In the following study, the effect of n-octanol on the conversion of TEOS in the EPMM membranes was investigated, and it was shown that nanocomposite membranes can be synthesized without any surfactant [45]. This was explained by the presence of ethanol, which is also a by-product of the hydrolysis and condensation of TEOS, and which is soluble in both the aqueous phase and the polymer solution phase. This study also revealed that although separating the hydrolysis and condensation reactions had led to greater conversions of TEOS, the glass transition temperature (T_g_) of the resulting two-step EPMM membranes did not change significantly compared to the reference PPO membrane. On the other hand, the single-step EPMM membranes (simultaneous hydrolysis and condensation reactions) showed lower TEOS conversions, but their T_g_ increased significantly compared to the reference PPO membrane [45]. This suggested a better dispersion of inorganic phase in the single-step EPMM membranes.

The objective of this paper is to investigate the impact of two different co-solvents, ethanol (EtOH) and acetone (Acet) on the properties of the resulting PPO-based EPMM membranes (EPMM/EtOH and EPMM/Acet) prepared in the single-step process. To maximize the conversion of TEOS, a parallel study was undertaken, in which silica nanoparticles were synthesized in a single-step process with pure solvent, trichloroethylene (TCE). The conversion of TEOS and the properties of the resulting silica nanoparticles were investigated. Then, by replacing pure TCE with a 10 *w*/*v*% solution of PPO in TCE while using the other conditions as established in the synthesis of silica nanoparticles, the EPMM membranes were synthesized and subsequently characterized by ^29^Si nuclear magnetic resonance (NMR) spectroscopy, Fourier transform infrared (FTIR) spectroscopy, inductively coupled plasma mass spectroscopy (ICP-MS), and contact angle measurements. The gas permeation properties of the synthesized membranes were studied with four different gases using a constant pressure (CP) system.

## 2. Materials and Methods

### 2.1. Materials

Poly(2,6-dimethyl-1,4-phenylene oxide) (PPO) powder with a molar mass of 350,000 g/mol was supplied by SABIC (Sittard, The Netherlands). Tetraethylorthosilicate (TEOS, reagent grade 98%), aluminum nitrate nonahydrate (98% A.C.S. reagent), sodium carbonate anhydrous (granular, A.C.S. reagent), trichloroethylene (TCE, 99.5% A.C.S. reagent), and ethanol (99%) were purchased from Sigma Aldrich (Oakville, ON, Canada). Acetone (99.5%) was purchased from Fisher Scientific (Ottawa, ON, Canada). All of the chemicals were used as received without further purification.

### 2.2. Synthesis and Characterization of Silica Nanoparticles

Silica powder was synthesized by the emulsion polymerization of TEOS using 1 M of aluminum hydroxonitrate solution, which played a role of a weak acid catalyst [46]. The aluminum hydroxonitrate solution, which is referred to as the aqueous solution, was prepared by slowly adding sodium carbonate solution (3.00 g of sodium carbonate in 13.0 mL of deionized water) into aluminum nitrite solution (10.50 g of aluminum nitrate in 15.0 mL of deionized water) [46]. The aqueous solution was magnetically stirred at room temperature (i.e., aged at room temperature) for different periods of time ranging from one to six days, before 1 mL of the aqueous solution was added into the oil phase (10 mL of TCE + 0.3 mL of either ethanol or acetone) followed by one-minute sonication of the content to form emulsion one. Emulsion two was formed by adding 0.3 mL of TEOS into emulsion one and sonication of the content for different periods of time ranging from 10 min to 40 min, during which the hydrolysis and condensation of TEOS took place simultaneously. Consequently, the sonication time of emulsion two is considered as a reaction time. The sonication of emulsions one and two were carried out using an ultrasonic homogenizer (Fischer Scientific, Model 550, Pittsburgh, PA, USA). Table 1 summarizes the preparation of W/O emulsions for the synthesis of silica powder.

After sonication of emulsion two, the content was transferred into a petri dish to let the volatile components evaporate at ambient temperature for at least 48 h. The remaining solids were post-treated to remove any unreacted TEOS and aluminum hydroxonitrate. The former was removed by dispersion in acetone followed by a four-hour centrifuging at 6000 rpm. After this, the remaining solids were re-dispersed in deionized water to remove any water soluble impurities, followed by another four hours of centrifuging at 6000 rpm and drying. Such post-treated solids are referred to as gravimetric powder method (GPM) powders. Apart from studying the effects of the aging time of the aluminum hydroxonitrate solution and the reaction time of emulsion two, the GPM tests also provided a guide for the synthesis of casting emulsions containing PPO, from which EPMM membranes were prepared.

The solid-state ^29^Si NMR spectroscopy (AVANCE 500, Bruker Biospin, Milton, ON, Canada) and FTIR spectroscopy (Agilent Cary 630 with DialPath technology, Mississauga, ON, Canada) were used to qualitatively verify the polymerization of TEOS in the emulsion and the formation of silica particles. Transmission electron microscopy (Tecnai F20 G2 FEI-TEM, Montreal, QC, Canada) was used to observe the morphology of the synthesized silica particles.

### 2.3. Synthesis and Characterization of Membranes

The protocol for the preparation of a membrane-casting emulsion is similar to the protocol used for the GPM with a couple of differences. First of all, a polymer solution (10 mL of 10 *w*/*v*% PPO solution in TCE) was used instead of a pure TCE for the preparation of emulsion one. In addition, emulsion two was sonicated for 90 min at the power level three compared to 10–40 min sonication at the power level seven in the case of the GPM protocol. The latter changes were dictated by a significantly greater viscosity of the polymer solution compared to the viscosity of pure TCE.

The sonicated emulsion two was used to cast the EPMM membranes by a spin-coating technique using a 100 series CEE Model Spinner. The details of the membrane casting protocol are described elsewhere [42,44,45]. The same casting protocol was also used to prepare the reference PPO membranes from a 10 *w*/*v*% PPO solution in TCE. The spin-coated EPMM and PPO membranes were post-treated in a two-step process consisting of: (1) boiling in deionized water for four hours to wash out all of the water-soluble residuals, and (2) heat treatment in nitrogen to remove the low-volatility and water-insoluble residuals. After the boiling step, some wrinkles appeared on the membrane surface, but the membranes remained transparent. The heat treatment was carried out at 235 °C, which is greater than the T_g_ of the PPO and EPMM membranes [45], and greater than the 120 °C that is used in the heat treatment of the first-generation EPMM membranes [44]. The heat treatment above T_g_ of the polymer ensures the removal of volatile residuals trapped in the Langmuir sites [47,48]. To ensure that the membranes remain transparent during the heat treatment step, the oven chamber had to be oxygen-free before increasing its temperature [45]. After the heat treatment step, the membranes shrank a little bit and became stiffer.

To verify the polymerization of TEOS and the removal of unreacted TEOS, the EPMM membranes were characterized using the solid-state ^29^Si NMR and FTIR spectroscopy. The membranes were also characterized in single gas permeation tests; the static water contact angle was measured using a VCA Optima Contact Angle System (AST Products, Inc., Billerica, MA, USA). The silica content was determined by an inductively coupled plasma mass spectroscopy (ICP-MS) using EPMM membrane samples. The measurements were performed on an Agilent HP 4500 ICP-MS machine (Mississauga, ON, Canada).

Single gas permeation tests with oxygen, nitrogen, methane, and carbon dioxide were carried out in a constant pressure (CP) system described elsewhere [42]. The experiments were performed at ambient temperature (22–23 °C) and feed pressures of ranging from 60 psig to 120 psig, while the permeate side of the membrane was open to atmosphere. To ensure reaching steady-state conditions, each test was carried out for at least 24 h. The permeability coefficient, P (Barrer), of a single gas in the membrane was then evaluated from:

(1) $$P=\frac{Qt}{A\left(p_{f}-p_{p}\right)}\times 10^{10}$$

where Q (cm^3^(STP)/s) is the steady state permeation rate, *t* (cm) is the membrane thickness, *A* (cm^2^) is the membrane area, and *p_f_* and *p_p_* are the feed and permeate pressures (cmHg), respectively. The selective properties of the membrane were evaluated by the ratio of the permeability coefficients determined from Equation (1), which is referred to as the ideal selectivity $\left(\alpha_{A/B}^{*}\right)$:

(2) $$\alpha_{A/B}^{*}=\frac{P_{A}}{P_{B}}$$

where subscript *A* refers to a more permeable gas, and subscript *B* refers to a less permeable gas.

## 3. Results and Discussion

### 3.1. Conversion of TEOS and Properties of Silica Powder

**Figure 1:**
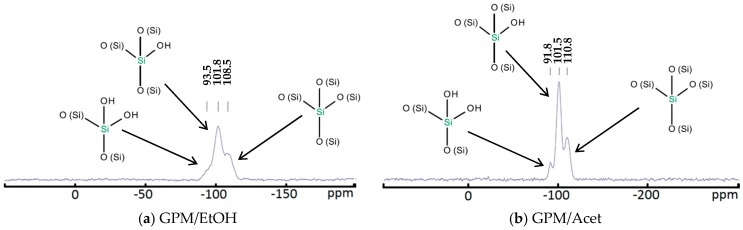
^29^Si NMR spectra of the synthesized powders: (**a**) GPM/EtOH and (**b**) GPM/Acet.

The net mass of dried powder is proportional to the conversion of TEOS; however, the actual TEOS conversion cannot be determined without the knowledge of the structure of the synthesized silica particles. Such information is provided by the ^29^Si NMR and FTIR analysis. Figure 1 presents the ^29^Si NMR spectra of the GPM/EtOH and GPM/Acet powders, respectively. In both cases, one can observe the peaks at −110 ppm, −100 to −104 ppm, and −90 ppm corresponding to the structural units Q^4^, Q^3^, and Q^2^, respectively [49,50]. The number in the superscript after Q indicates the number –O-(Si) bonds associated with a given Si atom. For both powders, the peak corresponding to Q^3^ is a dominant one; the intensity of this peak is slightly greater for GPM/Acet compared to GPM/EtOH, which might be indicative of a greater conversion of TEOS in the former powder. It is also important to emphasize that the absence of peaks other than Q^2^, Q^3^, and Q^4^ in Figure 1 indicates that there was no unreacted TEOS and TEOS dimer in the analyzed samples. Should the unreacted TEOS (Q^0^) and TEOS dimer (Q^1^) exist in the analyzed powders, the corresponding peaks at −82.5 ppm and −89.4 ppm would be observed [49].

**Figure 2:**
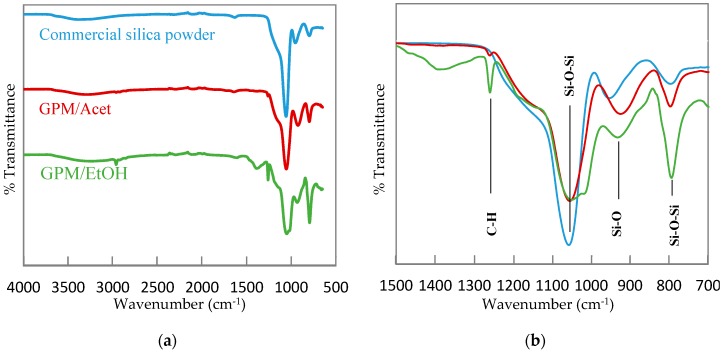
Fourier transform infrared (FTIR) absorption spectra for commercially available silica powder, GPM/Acet, and GPM/EtOH. (**a**) the entire spectrum; (**b**) the focused spectrum in 1500–700 cm^−1^ wavenumber range.

Figure 2 presents the FTIR spectra of the two synthesized silica powders, GPM/Acet and GPM/EtOH, which are compared to the FTIR spectrum of a commercial SiO_2_. The resemblance of the FTIR spectra of the synthesized silica powders to the spectrum of the commercial SiO_2_ is very apparent, which confirms the polymerization of TEOS in the presence of both co-solvents. More specifically, the formation of silica is confirmed by the absorption band appearing at around 1054 cm^−1^, which arises from the asymmetric stretch of the O atom in the Si–O–Si bridging bond [51]. Furthermore, the peak around 793–798 cm^−1^ corresponds to the Si–O–Si of the bending mode [52,53], while the band at 955 cm^−1^ can be assigned to Si-O lattice vibrations [54]. Unlike the commercial SiO_2_, the FTIR spectra of the synthesized silica powders, in particular GPM/EtOH, show one extra peak at 1259 cm^−1^, which corresponds to the symmetric deformation vibration of C–H bonds, which in turn represents the attachment of methyl groups to the Si atom [55]. Therefore, both ^29^Si NMR and FTIR qualitatively confirm the formation of silica by the emulsion polymerization of TEOS in pure TCE. The quantitative analysis of the conversion of TEOS, which are based on the mass of the formed dried powder, is discussed next.

The mass of GPM powders was measured following the previously described post-treatment of the solids remaining after the evaporation of emulsion two. Should there be no polymerization of TEOS, the remaining solids would be entirely aluminum nitrate and sodium carbonate, which were used for the preparation of the aluminum hydroxonitrate solution. This is because all of the other components in emulsion two were volatile. The evaporation of 0.1 mL of the aluminum hydroxonitrate solution, i.e., the amount used to form emulsion one, in a “dry run” test led to the average mass of solid residuals of 0.0309 g, which is very close to the theoretical value of 0.0305 g. In other words, any mass of the GPM powder greater than 0.0309 g is another indication of the actual polymerization of TEOS.

The quantitative assessment of the conversion of TEOS to silica based on the mass of the dried powder requires three assumptions: (i) the aluminum hydroxonitrate does not copolymerize with TEOS; (ii) dried powder does not contain residuals of aluminum nitrate and sodium carbonate; and (iii) the quantitative contributions of Q^2^, Q^3^, and Q^4^ are known. The first assumption can be justified by the aluminum hydroxonitrate solution playing only the role of a catalyst in the polymerization process of TEOS [45]. Considering the dispersion and centrifuging of the dried powder in deionized water, one can safely eliminate the presence of water-soluble components in the final dried powder. On the other hand, although it is evident from the ^29^Si NMR spectra in Figure 1 that Q^3^ is a dominating peak, the exact contributions of Q^2^, Q^3^, and Q^4^ are difficult to assess. Therefore, we considered three hypothetical cases, in which the polymerized TEOS is entirely in either Q^2^, Q^3^, or Q^4^ form to obtain a range of possible conversions of TEOS for a given mass of the dried powder.

Table 2 provides an example TEOS conversion ranges, using ethanol and acetone as the respective co-solvents, assuming three different forms of the polymerized TEOS. The GPM powders were prepared using the aqueous solution aged for four days, while emulsion two was sonicated for 30 min. The other experimental parameters are specified in Table 1. The mass of the final GPM/Acet dried powder (0.082 g) was greater than that of the dried GPM/EtOH powder (0.052 g). Consequently, depending on the assumed form of the polymerized TEOS, its conversion with acetone as a co-solvent ranges from 77.5% to 100%, while the conversion with ethanol as a co-solvent ranges from 54.8% to 71.2%. The details of the conversion calculations are shown elsewhere [42]. It can be noticed that for a given mass of the powder, the conversion increases from Q^2^ through Q^3^ to Q^4^. None of the conversion values shown in Table 2 is correct; however, considering that Q^3^ was a dominant peak in both GPM/Acet and GPM/EtOH spectra, and that the conversion based on Q^3^ peak falls in between those based Q^2^ and Q^4^ peaks, in the following discussion, the conversion of TEOS is based assuming that the polymerized TEOS was entirely in the Q^3^ form.

**Figure 3:**
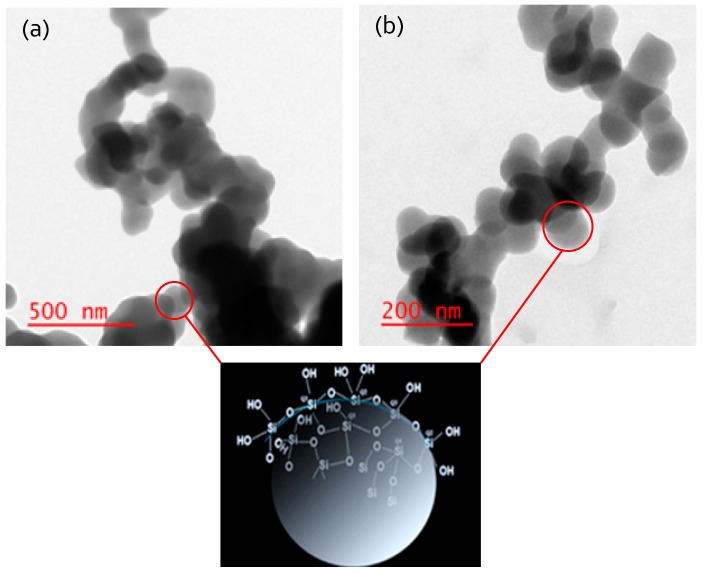
TEM images of silica particles (**a**) GPM/EtOH and (**b**) GPM/Acet.

A further insight on the effect of compatibilizer on the synthesized silica particles is provided by comparing the respective TEM images of the powders, which are presented in Figure 3. The use of an acid catalyst (aluminum hydroxonitrate) should lead to gel structures that are comparable to those from the original Stöber method. Indeed, it is evident from the micrographs in Figure 3 that for both ethanol and acetone co-solvents, synthesized silica particles have a spherical shape, which form linear structures; this morphology is suitable for the synthesis of polymer nanocomposites by the sol-gel or emulsion polymerized mixed matrix (EPMM) methods [56]. Keeping in mind that the scale in Figure 3a is greater than the scale in Figure 3b, the size of the synthesized silica particles in the presence of acetone, which ranges from 60 nm to 75 nm, is smaller than the 110–130 nm that was observed in the presence of ethanol. In fact, since the diameter of silica particles in the presence of acetone is less than 100 nm, they can be classified as nanoparticles.

### 3.2. Effect of Compatibilizer, Aging Time, and Reaction Time on the Conversion of TEOS in Silica Powder

**Figure 4:**
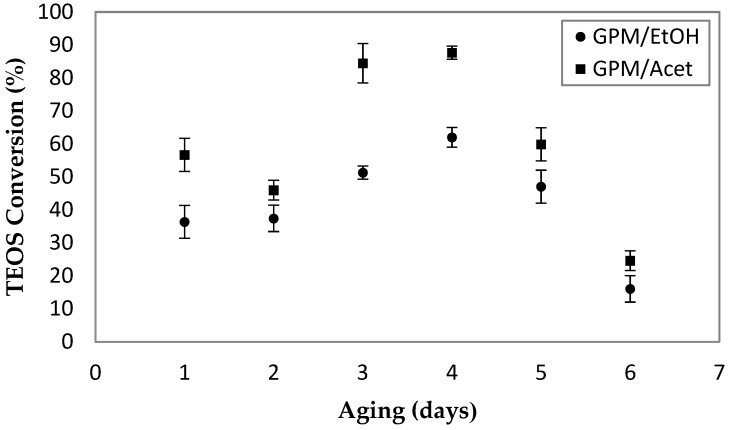
The effect aging time of aluminum hydroxonitrate solution on the conversion of TEOS. For each powder, emulsion two was sonicated for 30 min; all of the other synthesis parameters are provided in Table 1.

Figure 4 presents the effect of the aging time of the aluminum hydroxonitrate solution on the conversion of TEOS. All of the samples were prepared using the same 30-min sonication time of emulsion two. For each aging time, three samples of the powder were prepared, and the corresponding average values and standard deviations (error bars) are presented in Figure 4. The results indicate that four days of aging time, during which the aluminum hydroxonitrate solution was continuously stirred, leads to the maximum TEOS conversion for both co-solvents. It is important to note that regardless of the aging time, the pH of the aqueous solution was essentially constant, ranging from 3.6 to 3.8. For a given length of the aging time, the conversion of TEOS in GPM/Acet was greater than in GPM/EtOH. The difference between the two ranges from 10% to 30%. The reason behind the optimum aging time of four days is beyond the scope of this paper; nevertheless, this aging time was used to study the effect of the reaction time of emulsion two, and for the preparation of emulsion two containing PPO for the synthesis of EPMM membranes.

As already stated, the sonication time of emulsion two is equivalent to the actual reaction time, during which the hydrolysis and condensation reactions occur simultaneously. It was expected that by increasing the reaction time, the conversion of TEOS should increase. The actual effect of the reaction time on the conversion of TEOS in the presence of ethanol and acetone is summarized in Table 3. For each sonication time, three independent powder samples were prepared, and the resulting average and standard deviation values are reported in Table 3. It can be noticed that after a significant increase in the first 30 min, the following increase in the conversion was only marginal. The same trend is observed for both co-solvents. Hence, 30 min was considered as an optimum time for the sonication of emulsion two. As in the case of the effect of the aging time, for a given sonication time, the conversions of TEOS in GPM/Acet are 17–24% greater than those in GPM/EtOH.

The polymerization of TEOS, which is introduced into the system via TCE (i.e., the oil phase) requires the aluminum hydroxonitrate catalyst, which is present in the aqueous phase. Therefore, for the polymerization of TEOS to occur, a surfactant [44] and/or a co-solvent [45] is required. Acetone and ethanol, which were used in this study, are soluble in both water and TCE; however, the affinity of these co-solvents with the water phase and the oil phase are different. The difference in affinity can be quantified in terms of solubility parameters.

Table 4 presents the Hildebrand and Hanssen solubility parameters of water, TCE, ethanol, and acetone [57]. The closer the solubility parameters of the two components, the greater the affinity between them. The closeness of the Hildebrand solubility parameters is evaluated from:

(3) $$\Delta =\left|\delta_{i}-\delta_{j}\right|$$

where δ*_i_* and δ*_j_* are the Hildebrand solubility parameters of components *i* and *j*. In terms of the Hansen solubility parameters, the corresponding expression is given by:

(4) $$\Delta^{\prime }=\sqrt{{\left(\delta_{d,i}-\delta_{d,j}\right)}^{2}+{\left(\delta_{p,i}-\delta_{p,j}\right)}^{2}+{\left(\delta_{h,i}-\delta_{h,j}\right)}^{2}}$$

where δ*_d_*, δ*_p_*, and δ*_h_* are the dispersive, polar, and hydrogen bonding components of the Hansen solubility parameter, respectively, while subscripts *i* and *j* refer to the respective components; δ*_t_* is the total solubility parameter, which is calculated from the Hansen solubility parameters using:

(5) $$\delta_{t}=\sqrt{\delta_{d}^{2}+\delta_{p}^{2}+\delta_{h}^{2}}$$

The total solubility parameter is equivalent to the Hildebrand solubility parameter.

Considering the values of ∆ and ∆′, the affinity of ethanol to water is stronger than the affinity of acetone to water (∆_EtOH/W_ = 21.8 MPa^1/2^ and ∆′_EtOH/W_ = 24.0 MPa^1/2^ versus ∆_Acet/W_ = 28.3 MPa^1/2^ and ∆′_Acet/W_ = 35.7 MPa^1/2^). On the other hand, the affinity of ethanol to TCE is weaker than the affinity of acetone to TCE (∆_EtOH/TCE_ = 7.5 MPa^1/2^ and ∆′_EtOH/TCE_ = 15.4 MPa^1/2^ versus ∆_Acet/TCE_ = 1.0 MPa^1/2^ and ∆′_Acet/TCE_ = 7.9 MPa^1/2^). It is also important to keep in mind that if the polymerization of TEOS takes place, ethanol is also a by-product of both hydrolysis and condensation reactions [42]. Therefore, it is possible that on the one hand, the ethanol by-product acts as a “self-compatibilizer”, helping the conversion of TEOS, but on the other hand, because of its affinity to water, it may accumulate in the aqueous phase, shifting the hydrolysis and condensation reactions to the left. If acetone can initiate the polymerization of TEOS, and it is evident that it does, it might work better than the ethanol co-solvent, because ethanol is a by-product anyway. In addition, according to the differences in solubility coefficients, acetone might prefer to stay in the TCE-phase rather than in the aqueous phase; thus, helping to remove the ethanol by-product from the aqueous phase to the oil phase.

**Figure 5:**
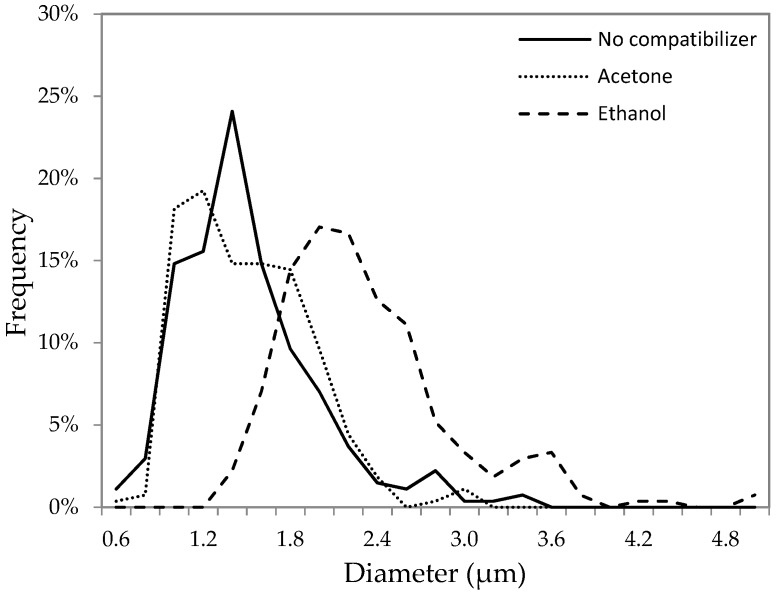
Size distribution of aqueous droplets in emulsion one with the poly(2,6-dimethyl-1,4-phenylene oxide) (PPO) solution in TCE as a continuous phase in the presence of different compatabilizers.

If ethanol accumulates in the aqueous phase, the size of the aqueous droplets in the presence of ethanol should be greater than in the presence of acetone. Due to a very low viscosity of the W/O emulsion formed with pure TCE, it was difficult to measure the size of the dispersed aqueous phase. On the other hand, these measurements were possible in more viscous emulsions, in which the oil phase contained PPO (10 *w*/*v*% solution of PPO in TCE). The size of the aqueous phase droplets in emulsion one was measured using an Olympus^®^ BX40 microscope and ImageJ software. Three different types of emulsion one were considered, without any co-solvent, and with ethanol and acetone as a co-solvent, respectively. The distribution of the droplet size in these three emulsions is presented in Figure 5. Each emulsion was sonicated for the same time (1 min), after which a sample from each emulsion was taken for the analysis. It is evident that the aqueous droplets in the presence of ethanol are greater than those in the presence of acetone. The average droplet diameter with ethanol as a co-solvent was 2.2 μm compared to 1.4 μm with acetone as a co-solvent. It is interesting to note that the droplet size distribution without any compatibilizer is similar to that in the presence of acetone. This confirms that acetone stays primarily in the oil phase, as concluded based on the ∆ and ∆′ values. Moreover, a greater size of droplets in the presence of ethanol might be responsible for a greater particle size in GPM/EtOH compared to GPM/Acet (Figure 3).

### 3.3. Conversion of TEOS in EPMM Membranes

Unlike the GPM, when PPO is present in the oil phase, after the casting of emulsion two and evaporation of volatiles, the main component in the resulting EPMM membrane is PPO. The synthesized silica particles (if any) are a minority component in the EPMM membranes. This makes the detection of silica in PPO-based EPMM membranes much more challenging than in GPM powders. To prove that the synthesized EPMM films contained silica particles, ^29^Si NMR and FTIR analyses were carried out using heat-treated films at 235 °C. Since 235 °C is greater than the T_g_ value of the PPO-based EPMM membranes [45], the heat treatment should facilitate the removal of any residual solvent and/or unreacted TEOS from the membranes

**Figure 6:**
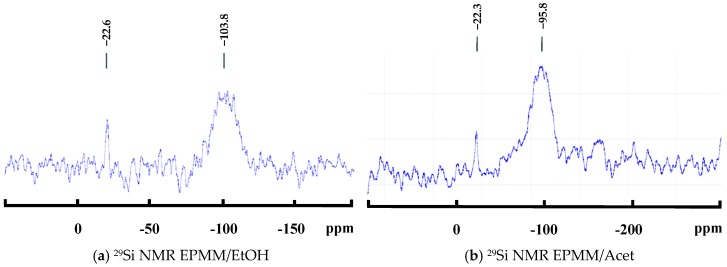
^29^Si NMR spectra of the synthesized emulsion polymerized mixed matrix (EPMM) membranes: (**a**) EPMM/EtOH membrane and (**b**) EPMM/Acet membrane.

The ^29^Si NMR spectra of the EPMM/EtOH and EPMM/Acet membranes are shown in Figure 6a,b, respectively. Both spectra show one broad peak at different locations: −103.8 ppm in EPMM/EtOH, and −95.8 ppm in EPMM/Acet. It is safe to assume that despite different locations, this broad peak originates from the overlapping of Q^2^, Q^3^, and Q^4^ peaks. At the same time, the difference in the location indicates different structures of the dispersed silica particles in the EPMM/EtOH and EPMM/Acet membranes. More specifically, the peak at −103.8 ppm suggests the domination of a branched Q^3^ structure in EPMM/EtOH, whereas the peak at −95.8 ppm suggests the domination of a linear Q^2^ structure in EPMM/Acet. The intensity of the broad peak in both membranes is small; it is not significantly greater than the oscillations of the baseline. It is interesting to note that in addition to the main broad peak, there is also one small, but sharp peak at approximately −22 ppm in both spectra, which cannot be attributed to any of the Si–O–Si bonds. This peak was not present in the spectra of the dried powders (Figure 1), and its origin is unclear. The absence of a peak at around −82.5 ppm, which would correspond to unreacted TEOS (Q^0^), confirms that 235 °C was sufficient to remove any unreacted TEOS from the membrane structure.

**Figure 7:**
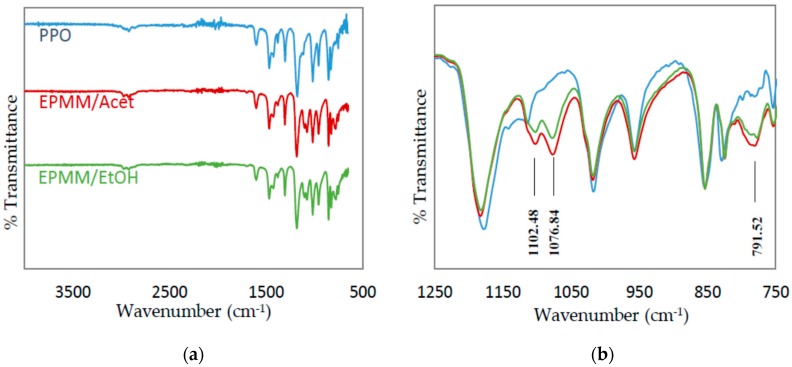
FTIR absorption spectra of the reference PPO, and EPMM/Acet and EPMM/EtOH membranes. (**a**) the entire spectrum; (**b**) the focused spectrum in 1250–750 cm^−1^ wavenumber range.

A further proof for the successful polymerization of TEOS in emulsion two is provided by the FTIR spectra. In addition to the spectra of the EPMM/Acet and EPMM/EtOH films, Figure 7 also presents the spectrum of a neat PPO film as a reference. The formation of silica particles in the EPMM membranes is confirmed by the presence of absorption bands at around 1102 cm^−1^ and 1076 cm^−1^ for Si–O–Si asymmetric stretching in linear and cyclic structures, respectively; in addition, the peak at around 780 cm^−1^ corresponds to Si–O–Si bending mode [58,59]. None of these peaks was present in the FTIR spectrum of the neat PPO. The intensity of these characteristic peaks appears to be slightly greater in EPMM/Acet than in EPMM/EtOH, which might indicate a greater conversion of TEOS in the former membranes. This is consistent with the ^29^Si NMR spectra shown in Figure 6, in which the intensity of the main broad peak relative to the peak at −22 ppm, as well to the oscillation of the baseline, appear to be greater in EPMM/Acet than in EPMM/EtOH.

To determine the silica content, a sample from a given membrane was first burned in a furnace at 450 °C for 16 h to remove the combustible (organic) portion; the produced ash was weighed and then digested in a mixture of HCl and HNO_3_ at 120 °C for 10 h, followed by diluting it with deionized water. Such a prepared sample was then analyzed for the Si and Al content using ICP-MS [42]. As expected, the Al peak was at a level of the instrument’s baseline, and only Si was detected. It is important to emphasize that regardless of the form of the synthesized silica in the EPMM membrane, it was assumed that the polymerized TEOS after the burning step was converted to the same final product, SiO_2_, i.e., the Q^2^ form. Consequently, the conversions of TEOS reported in Table 5 are the lowest possible values, while the inorganic loading values are provided for the three hypothetical cases, which are similar to GPM powders reported in Table 2. Should all of the TEOS added to emulsion two be polymerized, the inorganic loading in the resulting EPMM would vary depending on the silica structure, from 7.52% for the entirely linear (Q^2^) form to 9.57% for the totally branched (Q^4^) form. The numbers shown in Table 5 are the average and the corresponding standard deviation values from four samples coming from two membranes, i.e., each membrane was cut in half to generate two samples, which were then processed as described above.

The average conversions of TEOS in the presence of ethanol and acetone were 29.6% and 35.7%, respectively. This is qualitatively consistent with the effect of co-solvent on the conversion of TEOS in the GPM powders. In other words, the use of acetone as a co-solvent leads to greater conversions of TEOS, which can be explained on the basis of a stronger affinity of acetone compared to ethanol to the oil phase, and the accumulation of ethanol in the aqueous phase. The results in Table 5 also confirm what was qualitatively observed in Figure 6 and Figure 7 based on the intensity and the size of the relevant peaks in these figures. At the same time, it is important to note that the conversions of TEOS in emulsion two when the oil phase is a PPO solution in TCE are less than half the values when the oil phase is pure TCE. On the one hand, a viscous polymer solution might be improving the stability of emulsion two, which would favor greater conversions of TEOS, but on the other hand, the diffusion coefficient of TEOS in a viscous solution of PPO in TCE must be significantly smaller than that in a non-viscous pure TCE, thus reducing the conversion of TEOS. Therefore, it is evident that in the case of this study, the latter was more prominent than the former.

### 3.4. Gas Transport Properties of EPMM Membranes

The average contact angels of distilled water on the surface of the neat PPO, EPMM/EtOH, and EPMM/Acet membranes along with the corresponding standard deviation values are shown in Table 5. It can be noticed that the average contact angle decreases from 77.7° for PPO to 73.2° for EPMM/Acet. This could be expected, since silica is more hydrophilic than PPO. On the other hand, the observed increase in the hydrophilicity of the EPMM membranes is very small, because of the relatively small corresponding inorganic loading.

Table 6 summarizes the gas permeation properties of the reference PPO, EPMM/EtOH, and EPMM/Acet membranes determined in single-gas permeation experiments. The reported permeability coefficients were calculated using Equation (1) based on the measured steady-state permeation rates of the respective gases. The values in Table 6 are the average and the corresponding standard deviations from at least three membranes of the same type.

In comparison to the reference PPO, the EPMM membranes are considerably more permeable. The greatest increase in gas permeability (c.a. 2.5 times) is observed for CH_4_, which is followed by CO_2_, for which the permeability coefficient nearly doubled. The permeability coefficients of O_2_ and N_2_ increased by roughly 50%. The EPMM/Acet membranes are both more permeable and more selective than the EPMM/EtOH membranes. This can be attributed to a slightly greater conversion of TEOS in the former membranes. However, the main reason is probably the different structure of the polymerized TEOS in these membranes. As already discussed, based on the ^29^Si NMR spectra presented in Figure 6, the polymerized silica in EPMM/EtOH is mainly in a branched form, while in EPMM/Acet, it is mainly in a linear form.

One of the biggest challenges associated with the dispersion of inorganic particles in a continuous phase of an organic polymer is weak interactions between the two phases, which may lead to the agglomeration of inorganic particles and eventually a phase separation. If the latter occurs, an increase in gas permeability is associated with a significant loss in gas selectivity. On the one hand, considering *α*(CO_2_/CH_4_), the ideal selectivity of EPMM membranes decreased compared to the reference PPO membrane, but on the other hand, considering *α*(O_2_/N_2_), the ideal gas selectivity of EPMM membranes is actually slightly greater. This provides an indirect proof that synthesized silica particles are well dispersed in the PPO matrix. Moreover, although we were not able to measure the size of the dispersed silica particles in the EPMM membranes, it can be hypothesized that they were much smaller than those observed in GPM powders (Figure 4). Since the silica particles and PPO chains do not interact chemically, strong physical interactions between silica particles and PPO chains require the former to be very small. The hypothesized smaller size of silica particles in EPMM membranes compared to the GPM powders can also be justified by a much greater viscosity of the PPO solution compared to the viscosity of pure solvent. The high viscosity of the continuous oil phase could have prevented the agglomeration of the dispersed aqueous droplets, thus limiting the size of the polymerized TEOS.

The transport in EPPM membranes can be assumed to follow a solution–diffusion model, in which the permeability coefficient is a product of the diffusion coefficient and the solubility coefficient. The observed increase in the gas permeability of the EPMM membranes compared to the reference PPO membrane is due in part to the increase in the free volume, because nanosized inorganic fillers are known to disrupt molecular chain packing, thus acting as spacers, which create additional volume for diffusing gases [60,61]. However, that the permeability coefficients of the different gases increased by markedly different factors suggests that dispersed silica nanoparticles must also affect the solubility coefficients, in particular those of CO_2_ and CH_4_, in the EPMM membranes. Considering that CO_2_ and CH_4_ have considerably greater affinity toward silica than N_2_ and O_2_, a greater increase in the permeability coefficients of CO_2_ and CH_4_ compared to N_2_ and O_2_ could be explained on the basis of an increase in the solubility coefficients of CO_2_ and CH_4_ in EPMM membranes. Although the affinity of CO_2_ to silica is greater than that of CH_4_, the permeability coefficient of CH_4_ increases relatively more than the permeability coefficient of CO_2_, which is why the ideal CO_2_/CH_4_ selectivity of the EPMM membranes is smaller than the ideal CO_2_/CH_4_ selectivity of the reference PPO membranes. One possible explanation arises from CO_2_ being a smaller molecule than CH_4_, and therefore, an increase in the diffusion coefficient of CO_2_ due to the additional free volume created by the dispersed nanoparticles, is greater than the corresponding increase in the diffusion coefficient of CH_4_.

**Figure 8:**
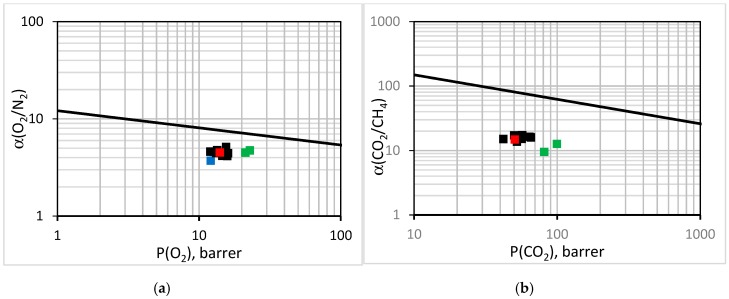
Performance of the synthesized EPMM (green squares) membranes, reference PPO (red square) membranes, and first-generation EPMM membranes [43] (blue square) in comparison to the literature data for PPO [29,30,31,32,33,34,37,59,60,61,62] (black squares) and the upper bound line for (**a**) O_2_/N_2_ and (**b**) CO_2_/CH_4_.

To assess the gas transport properties of membranes, they are customarily compared to the upper bound line for a given pair of gases [62]. The performance of the EPMM and the reference PPO membranes is compared with the literature data for PPO [30,31,32,33,34,35,38,63,64,65,66] and the first-generation EPMM membranes [44] in Figure 8. Considering O_2_/N_2_ (Figure 8a) and CO_2_/CH_4_ (Figure 8b), all of the data points in Figure 8 are below the respective upper bound lines. The distance to the upper bound line is smaller for O_2_/N_2_ than for CO_2_/CH_4_. The literature data for PPO is not a single point in Figure 8, because the intrinsic gas transport properties of polymeric membranes are known to be affected by the molecular weight of the polymer [63,67] and the solvent that was used to form the casting solution [68]. It is evident that the reference PPO membrane falls well within the literature points. Although the EPMM membranes that were synthesized in this study are below the upper bound line, for O_2_/N_2_, they move closer to this line compared to the reference PPO, because the increase in O_2_ permeability is associated with the practically unchanged ideal O_2_/N_2_ selectivity. In contrast, the first-generation EPPM membranes are both less permeable and less selective not only compared to the new EPMM membranes, but also to the reference PPO. Considering the CO_2_/CH_4_ performance, the new EPPM membranes are not any closer to the upper bound line compared to the reference PPO; doubling of the CO_2_ permeability coefficient is associated with a decrease in the ideal selectivity of CO_2_/CH_4_.

## 4. Conclusions

The new PPO-based EPMM membranes, in which TEOS was polymerized in an aqueous phase dispersed in a continuous phase of polymer solution, were successfully synthesized. Since TEOS is only partly soluble in water, the effect of the two different co-solvents (ethanol and acetone) added to the oil phase was investigated. The new EPPM membranes were synthesized without using any surfactant. To optimize the conditions for the polymerization of TEOS, a parallel investigation in which the continuous phase was pure TCE, was carried out. This parallel investigation revealed greater conversions of TEOS in the presence of acetone, as well as the structure and the size of the synthesized silica nanoparticles. The corresponding EPPM membranes that were synthesized in the presence of acetone also showed a greater conversion of TEOS and a different structure of dispersed silica nanoparticles compared to the EPMM membranes synthesized in the presence of ethanol. Both types of EPMM membranes were considerably more permeable to gases compared to the reference PPO membrane. Although the CO_2_/CH_4_ ideal selectivity decreased, the O_2_/N_2_ ideal selectivity of the new EPMM membranes was comparable to that of the reference PPO. As a result, the new EPPM membranes, in particular those synthesized in the presence of acetone, moved closer to the O_2_/N_2_ upper bound line.

Although the current study was limited to PPO–silica nanocomposite membranes, the EPMM method can be used to synthesize other types of nanocomposite membranes in which synthesized nanoparticles do not need to chemically interact with the organic polymer. Moreover, the current study provides a guide on the selection of a co-solvent based on its solubility parameter.

## Figures and Tables

**Table 1:** Preparation of the water/oil (W/O) emulsions for the synthesis of silica powder. Acet: acetone, EtOH: ethanol, GPM: gravimetric powder method, TCE: trichloroethylene, TEOS: tetraethylorthosilicate.

Powder Code	Emulsion One = 10 mL of TCE + 0.1 mL of Aqueous Solution ^1^ + Additives				Emulsion Two = Emulsion One + 0.3 mL of TEOS	
	Additives		Sonication ^2^		Sonication ^2^	
	EtOH (mL)	Acetone (mL)	Power Level	Time (min)	Power Level	Time (min)
GPM/EtOH	0.3	-	7	1	7	10–40
GPM/Acet	-	0.3	7	1	7	10–40

^1^ Aluminum hydroxonitrate 1 mol/L solution; pH: 3.6–3.8; aging time: one to six days. ^2^ Ultrasonic homogenizer: Fischer Scientific, Model 550.

**Table 2:** Hypothetical TEOS conversions based on the mass of dried powder.

Powder Code	Net Powder Mass	TEOS Conversion		
		Q^2^	Q^3^	Q^4^
GPM/EtOH	0.058	54.8%	62.0%	71.3%
GPM/Acet	0.082	77.5%	87.6%	100%

**Table 3:** The effect of the sonication time of emulsion two on the TEOS conversion. Aqueous solution was aged for four days in each case; all of the other synthesis parameters are provided in Table 1.

Powder Code	Emulsion One				Emulsion Two		TEOS Conversion (%)
	Compatibilizer		Sonication		Sonication		
	EtOH (mL)	Acet (mL)	Power level	Time (min)	Power level	Time (min)	
GPM/EtOH-A	0.3	-	7	1	7	10	35 ± 5
GPM/EtOH-B	0.3	-	7	1	7	20	53 ± 4
GPM/EtOH-C	0.3	-	7	1	7	30	62 ± 2
GPM/EtOH-D	0.3	-	7	1	7	40	64 ± 4
GPM/Acet-A	-	0.3	7	1	7	10	52 ± 5
GPM/Acet-B	-	0.3	7	1	7	20	66 ± 3
GPM/Acet-C	-	0.3	7	1	7	30	88 ± 3
GPM/Acet-D	-	0.3	7	1	7	40	89 ± 2

**Table 4:** Hildebrandt and Hansen solubility parameters of the aqueous phase (water), the oil phase (TCE), and two compatabilizers: ethanol and acetone [57].

Solvents	$\delta_{d}$	$\delta_{p}$	$\delta_{h}$	$\delta_{t}$	δ
	MPa^1/2^	MPa^1/2^	MPa^1/2^	MPa^1/2^	MPa^1/2^
TCE	18.0	3.1	5.3	19.0	18.7
water	15.6	16.0	42.3	47.8	48.0
ethanol	15.8	8.8	19.4	26.5	26.2
Acetone	15.5	10.4	7.0	20.0	19.7

**Table 5:** Contact angle, conversions of TEOS, and inorganic loadings in the membranes.

Membrane	Contact Angle	TEOS Conversion (%)	Inorganic Loading (wt %)		
			Q^2^	Q^3^	Q^4^
PPO	77.7 ± 2.1	-	-	-	-
EPMM/EtOH	76.9 ± 1.8	29.6 ± 0.6	3.0 ± 0.1	2.7 ± 0.1	2.3 ± 0.1
EPMM/Acet	73.2 ± 2.5	35.7 ± 0.8	3.6 ± 0.1	3.2 ± 0.1	2.8 ± 0.1

**Table 6:** Single gas permeabilities and ideal selectivities of the reference PPO and EPMM membranes.

Membrane	*P*_*O*_2__	*P*_*N*_2__	*P*_*CH*_4__	*P*_*CO*_2__	*α*(O_2_/N_2_)	*α*(CO_2_/CH_4_)
	(Barrer)	(Barrer)	(Barrer)	(Barrer)	(-)	(-)
PPO	14.0 ± 2.1	3.1 ± 0.6	3.4 ± 0.2	50.5 ± 3.3	4.5	14.9
EPMM/EtOH	21.2 ± 3.1	4.7 ± 0.7	8.5 ± 0.6	81.2 ± 4.1	4.5	9.5
EPMM/Acet	22.8 ± 4.9	4.8 ± 1.2	7.9 ± 1.4	99.6 ± 8.3	4.8	12.7
